# Prevalence, intensity and associated risk factors of soil transmitted helminth infections: A comparison between Negritos (indigenous) in inland jungle and those in resettlement at town peripheries

**DOI:** 10.1371/journal.pntd.0007331

**Published:** 2019-04-22

**Authors:** Azdayanti Muslim, Sakinah Mohd Sofian, Syahrul Azlin Shaari, Boon-Peng Hoh, Yvonne Ai-Lian Lim

**Affiliations:** 1 Department of Parasitology, Faculty of Medicine, University of Malaya, Kuala Lumpur, Malaysia; 2 Department of Medical Microbiology and Parasitology, Faculty of Medicine, Universiti Teknologi MARA (Sungai Buloh Campus) Sungai Buloh, Selangor, Malaysia; 3 Institute of Medical Molecular Biotechnology (IMMB), Faculty of Medicine, Universiti Teknologi MARA (Sungai Buloh Campus), Sungai Buloh, Selangor, Malaysia; 4 Faculty of Medicine & Health Sciences, UCSI University Kuala Lumpur Campus, Cheras, Kuala Lumpur, Malaysia; 5 Centre of Excellence for Research in AIDS (CERiA), University of Malaya, Kuala Lumpur, Malaysia; Consejo Nacional de Investigaciones Cientificas y Tecnicas, Fundación Mundo Sano, ARGENTINA

## Abstract

**Background:**

Formerly known as the Malaysian hunter gatherers, the Negrito Orang Asli (OA) were heavily dependent on the forest for sustenance and early studies indicated high prevalence of intestinal parasitism. Initiation of a redevelopment program in the 1970s aimed to demarginalize the OA was expected to reduce soil transmitted helminth (STH) infections. Gradually, the OA were relocated to new resettlement areas at the peripheries. The aim of this study was to compare STH infections between Negritos who are still living in the inland jungle with those living in resettlements.

**Methodology/Principal findings:**

A total of 416 Negrito participants were grouped into two categories of communities based on location; Inland Jungle Villages (IJV); and Resettlement Plan Scheme (RPS). Iodine wet mount, formalin-ether sedimentation, modified Trichrome and modified Ziehl-Neelsen staining and Kato-Katz methods were performed on stool samples. A questionnaire was used to collect information regarding demographic, socioeconomic, environmental and hygiene behaviors. Prevalence of STH was significantly higher in IJV (91.3%) versus RPS (83.1%) (P = 0.02). However, the percentage of individuals with severe intensity of *Trichuris trichiura* infections was significantly higher in the RPS (17.2%) compared to IJV (6.5%) (P = 0.01). Severe *Ascaris lumbricoides* infection was observed at 20.0% amongst RPS Negritos and 15.0% amongst IJV (P = 0.41). Whilst for hookworm infection, both prevalence and individuals with moderate to severe infections were higher in the IJV (26.2%, 41.0%) versus RPS (18.7%, 24.0%) (P values = 0.08, 0.09), accordingly. The prevalence other intestinal parasitic infections (e.g. *Entamoeba* sp., *Blastocystis* and flukes) was also higher in IJV versus RPS. Apart from poor hygienic behaviors as significant risk factors in both communities, low socio-economic status was highly associated with STH infections in RPS (P<0.001) but not significantly associated in IJV.

**Conclusions:**

The findings showed that *ex situ* development plan by RPS has not profoundly contributed to the STH reduction among the OA. Conversely, burden rate of *T*. *trichiura* infections increased due to their extreme poverty and poor hygienic behaviors. Here, we are suggesting biannual mass albendazole intervention (triple dose regimens in RPS, but a single dose in IJV) and community empowerment to both communities. For a long-term and better uptake, these strategies must be done together with the community input and participation, respecting their traditional customs and accompanied by recruitment of more OA people in the health-care taskforce.

## Author summary

Pattern of diseases in a community are associated with changes of culture, socio-economic and environmental conditions. In Malaysia, while transition towards modernity by socio-economic development has shown significant reduction in intestinal parasitism in the general population, no significant difference was observed among the Orang Asli (indigenous) communities. Nevertheless, since the 1970s, the Malaysian government has initiated the Resettlement Plan Scheme (RPS) to improve health, socio-economic status and education opportunities of the OA. Known as hunter gatherers, the Negritos represent a good model to study the effects of living conditions transition on intestinal parasitism. Like other tribes, they are also undergoing the transition from foraging and gathering practices towards demarginalization primarily by resettlement. Thus, the present study aims to evaluate STH infections in two categories of Negritos; (i) Inland Jungle Villages (IJV) communities, living in the interior forested areas and (ii) the Negritos who have been gathered and relocated to designated settlements closer to towns (RPS). A study to compare the current pattern of STH profiles in terms of prevalence and intensity of infection together with their associated risk factors among these two categories of communities was conducted. Since STHs are still plaguing the OA communities, the information gathering in the present study will be crucial for customizing a cost-effective deworming program involving selection of drug dosages, frequency of administration, type of modalities and most importantly community inclusion and empowerment programs through health education.

## Introduction

Cultural, socio-economic and environmental changes affect the pattern of diseases and health persistence within a population [1]. According to palaeoparasitology history, intestinal parasites, particularly soil-transmitted helminths (STH) had co-evolved together with humans since the very beginning, as was proven by the discovery of the parasites in an ancient fecal sample [2]. As time passed, the colonization of intestinal parasites has declined tremendously in certain parts of the world, greatly influenced by the rapid improvement of socio-economic status and sanitation standards, as seen in developed nations [3]. In contrast, however, owing to the lack of proper sanitation and good hygiene practice, intestinal parasitic infections (IPI) are still afflicting a majority of the deprived and underserved peoples, for example the indigenous populations in low- and middle-income countries [4, 5].

In Malaysia, the Orang Asli (OA) indigenous (which means the ‘original people’ or ‘the first people) population has experienced a state of transition between hunter-gatherer lifestyles with strong adherence with the tropical rain forest towards modernity. The transition took place since the initiation of a redevelopment program among OA communities in 1970s by the Malaysian government, with the aim to demarginalize the OA [6, 7]. The program was primarily conducted by improving existing OA villages (*in situ* development) and initiating resettlement plans (Rancangan Pengumpulan Semula or RPS) (*ex situ* development). Under the RPS program, the OA were regrouped and relocated to new resettlements at the peripheries or near township areas with the aim to improve their health by having better access to health-care facilities, increasing their socio-economic status and education opportunities. Basic facilities such as housing, electricity and water supply, and schools were provided. Customary cultures such as foraging, hunting and subsistence cropping were gradually replaced by cash crop agriculture (e.g. palm oil or rubber plantations). These RPS communities consequently developed a closer contact with other mainstream communities (e.g. the Malay community) [6, 7]. In 2011, the fraction of the OA communities that have been relocated under the RPS program was 63.0% [8, 9]. On the other hand, some tribes of OA still live and remain in the inland within the forest areas (37.0%) and still prefer the foraging and gathering lifestyles despite provision of basic infrastructure in their villages (*in situ* development) [7, 9].

While the transition towards modernity by the rapid growth of socio-economic advancement has shown significant reduction of STH in the general population, such improvement is negligible among the OA communities [10, 11], since STH infections are still highly endemic [12–14]. Given these contrasting findings, the pre-assumptive idea that demarginalization and redevelopment among OA lead to a better standard of living and consequently facilitate the reduction of intestinal parasitism among this community need to be reevaluated [10, 15]. While most of the prevalence studies highlighted low household income and sanitary behavior as the significant risk factors for STH infections among OA [12, 16, 17], very few focused on the effect of demarginalization and resettlement on the infections. Therefore, our ultimate objective is to address the latter aspect, which could be achieved primarily by comparing the findings from two different OA communities from similar tribes which have different environment and ecological settings.

In the present study, from three OA tribes; Senoi, Proto-Malay (or Aboriginal-Malay) and Negrito [18, 19], the Negrito was chosen as our study population based on three aspects; the tribe is the earliest inhabitants in Peninsular Malaysia (~50,000 years ago) [20], have the least population (~5000) currently and are known as hunter gatherers [19]. Moreover, except for a few reports available on the Negritos, the majority of the STH infection studies were conducted mainly among other tribes, namely the Senoi and Proto-Malay due to their larger population sizes [16, 21, 22]. Given these factors, we believed the Negritos provide a good model in determining how transition towards demarginalization influences the pattern of STH infections that traditionally inflict them [21].

Here, we report a finding pertaining on the current pattern of intestinal parasitism, STH infection intensity and associated risk factors by comparing the Negritos who are still living in the inland jungle (*in situ* improvement) and those who have undergone resettlement (RPS *ex situ* development) at the peripheries of towns.

## Materials and methods

### Ethical approval

This study received ethical approval from both the Ethics Committee of the Universiti Teknologi MARA (reference no: 600- IRMI (5/1/6) and National Medical Research Register (NMRR), Ministry of Health, Malaysia (NMRR-17-3055-37252 (IIR). Permission to conduct the study was obtained from the Department of Orang Asli Development (JAKOA) [Reference no: JAKOA/pp.30.052J1d9 (29)].

### Study design, sample size calculation and study population

A cross-sectional survey with a convenient, snowball sampling method was carried out from May 2016 to April 2017 in eight villages comprising all six Negrito sub-tribes; Jahai, Bateq, Kintak, Lanoh, Kensiu and Mendriq. The villages were selected based on the list of Negrito villages permitted by JAKOA, by further invitations from headman of the tribes, and after agreement and willingness of participation by each headman and members of their village. These villages are all located in the northern states of Peninsular Malaysia (S1A Fig). Prior to sample collection, visits and meetings were conducted at the selected villages. A short briefing was held before each sampling session, during which the purpose and method of the study were clearly informed to prospective participants. Those who agreed to participate either signed or thumb-printed informed consents, witnessed by the accompanying JAKOA officer(s). Permission and consent via signature from legal representatives were obtained for participants below 12 years old. Participants were informed of their rights to withdraw from the study at any time without prior notice.

For the purpose of comparison, the villages were grouped into two categories based on location and types of development; ‘Inland Jungle Villages (IJV) of *in situ* improvement’ (S1B Fig) and ‘Resettlement Plan Scheme (Rancangan Pengumpulan Semula or RPS) of *ex situ* development’ (S1C Fig). The IJV community refers to the group of Negritos staying in inland tropical rain forest (as in the case of Lanoh, Kintak and some groups of Batek and Jahai) with some still practicing hunting and gathering lifestyles. Each village has a population size from as low as 50 to as high as 300 individuals and were not easily accessible, except by boat and four-wheel-drive vehicles. Despite being officially recognized by the government, the villages lacked basic facilities, some lacking electricity due to their very remote locations. Because contact with outsiders was minimal, their transition towards demarginalization is regarded as slow.

Meanwhile, the RPS community is defined as groups of Negritos which have gathered and relocated into designated settlements at the peripheries of towns (these Kensiu, the Mendriq and some groups of Batek and Jahai) by JAKOA. Each resettlement has a population size of between 170 and 450 people. Their locations are near the road, easily accessible and their communities are provided with better housing, piped water supply, electricity, education and health-care facilities. Their transition towards assimilation and demarginalization with mainstream populations within the country is faster. A detailed summary of the sub-tribes and villages included in this study is shown in Table 1.

**Table 1:** Number of samples with the estimated number of Negritos based on their sub-tribes and community categories in Malaysia.

Sub-tribes	Village, state	Community category		N (%)	*Estimated total population in Malaysia
		IJV^¥^ n	RPS^β^ n		
**Jahai**	RPS Banun, Perak Kg Bunga Hilir, Perak	0 13	89 0	102 (4.6)	2,195
**Bateq**	Kuala Tahan, Pahang Kg Sg Berjuang, Pahang	12 0	0 45	57 (3.1)	1,842
**Kensiu**	Kg Ulu Legong, Kedah	0	91	91 (36.3)	251
**Lanoh**	Kg. Air Bah, Perak	47	0	47 (13.1)	359
**Kintak**	Kg. Bukit Asu, Perak	77	0	77 (47.5)	162
**Mendriq**	Kg. Kuala Lah, Kelantan	0	42	42 (21.0)	200
	**Total N (%)**	149 (35.8%)	267 (64.2%)	416 (8.3%)	~5,009

*Estimated population based on JAKOA’s 2010 Population and Housing Census [24]

^¥^IJV (Inland jungle villages) = These villages are located in remote area of tropical rain forest. Their population have been officially recognized by the government and included under *in situ* improvement program. They have access to the forest ecosystem for hunting and gathering forest resources due to their vicinity within the forest.

^β^ RPS (Resettlement Plan Scheme) = The Negritos that have been re-grouped and relocated into new resettlement areas as designated by JAKOA. Their new resettlement areas are located at the peripheries of the town, near to the road and closer to dominant population in Malaysia (*ex situ* development)

The sample size for this study was determined according to the formula by Wang and Chow [23]. The calculation was performed using PS Power and Sample Size Calculation Software for two proportions with the following parameters; 5% level of significance at 95% confidence interval, 80% power of study and anticipated STH infection prevalence as follows: 81.3% among RPS [16] and 96.5% among IJV Negritos [21]. By adjusting for 25% attrition rate, the minimum number of participants required in this study was estimated at 160 (80 participants from each population category).

We managed to voluntarily recruit a total of 430 Negritos in this study. However, only 416 of these (representing approximately 8.3% of the entire Negrito population in Malaysia) which had paired stool samples and datasets were included in the analyses (Table 1). Out of the 416 participants, 149 (35.8%) Negritos were categorized under IJV and the remaining 267 (64.2%) belonged to RPS.

### Questionnaire

Interviews were conducted by two trained research assistants in the Malay language based on a pretested questionnaire. The questions were mainly on participants’ demographic data (i.e. sub-tribes, age, gender and number of family members), socio-economic and educational status (i.e. household monthly income, occupational status and level of education), general sanitation and environmental conditions, which include source of water supply and availability of latrine system (pour/flush toilet or pit latrine). The source of water supply was categorized into treated (government piped water) and untreated (river, lakes, mountain water, etc.). Other questions were on behavioral risks (personal hygiene) such as habit of washing hands after defecation or after playing/in contact with soil, indiscriminate defecation (preference to defecate anywhere without any specific location or latrine, usually in the bushes or near the river), and closer contact (own or always playing) with domestic animals (commonly dogs and cats). Finally, we also asked about their previous history of anti-helminthic treatment; whether they have taken any anti-helminthic drug (prescribed by medical personnel or during a deworming program) or none at all during their entire life. For young children, the information was collected by interviewing their parents or guardians in their home setting.

### Stool sample collection and parasitological examination

Those with a history of antibiotic or anti-helminthic treatment in the previous 3 months before the commencement of the study were excluded from this study. Pre-labelled capped stool containers were given to the participants a day before stool sample collection along with instruction on correct placement of their stool into the containers. Stool samples were collected without age discrimination, the following morning between 8 and 11 am. Each stool sample was divided into three aliquots [fresh, 10% formalin (only a few drops of formalin so that the samples do not became liquefied), and 2.5% potassium dichromate], sealed with zip-locked plastic bag, kept in cool boxes during transportation, transferred to a laboratory in the Institute for Medical Molecular Biotechnology (IMMB), Universiti Teknologi MARA within 6 to 12 hours of duration and stored at 4 °C before examination.

The laboratory examination was conducted using different methods as soon as possible upon the arrival of samples to the laboratory. The samples were first processed using a standard direct wet smear and formalin–ether sedimentation [25] to detect the presence of ova or cyst in the stool samples. A modified Kato–Katz method was then applied to quantify the burden of STH infections within 4 hours of reaching the laboratory. Duplicate 41.7 mg Kato–Katz thick smears were prepared from each fresh and formalin-fixed sample (the preservative was discarded first), and were read twice after 15 minutes by two different technologists. The corresponding results were compared and in cases of significant inconsistencies (positive vs. negative and/or difference in egg counts of more than 20%), the slides were re-examined. The total number of eggs was multiplied by a factor of 24 (number of eggs × 24) to produce the number of eggs per gram (epg) of feces. The lower limits of moderate and heavy infections were 5,000 and 50,000 epg for *A*. *lumbricoides*; 1,000 and 10,000 epg for *T*. *trichiura*; and 2,000 and 4,000 epg for hookworm [26, 27]. Modified Wheatley Trichrome (according to manufacturer’s protocols—Thermo Scientific Remel Trichrome Stain Kit) and modified acid-fast staining techniques (adapted from CDC DPDx stool specimens—staining procedure) were also incorporated in this study to facilitate detection and provide accurate identification of intestinal protozoa. Stool samples were considered positive if the intestinal parasites were detected by any of these methods.

### Data management and statistical analysis

Data were double-entered, cross-checked and merged into a single data set in a Microsoft Office Excel 2010 document. Statistical analysis was performed using IBM SPSS version 20 (SPSS, Chicago, IL, USA). Demographic data, socio-economic, environmental and behavioral factors were treated as categorical variables. For the number of family members, we categorized them into <7 members and >7 members as it was generally common among OA to have large family sizes. Monthly household income was categorized into ~ <USD 125 and >USD 125 because more than half of the studied populations have a gross monthly income of less than USD 125. Descriptive statistical analyses were performed to obtain a clear understanding of the population. Frequency, percentages (rate), measures of central tendency (means, medians and other percentiles) and dispersion (standard deviations, ranges) were computed to describe the characteristics of the studied population. Shapiro–Wilk statistic coupled visual diagrams (Q–Q Plots and histogram) were used for assessing the normality of the scores. P-value of more than 0.05 indicates normality.

For normally distributed data, arithmetic means with standard deviations (SD) or standard error of the mean (SE) were presented. For data not normally distributed, medians with data range (interquartile, IQR) were used. Pearson’s chi-squared test (*χ*^2^) or Fisher’s exact test were used to determine the independence between categorical independent variables and the outcome. The same test was used to measure the association of STH infections with the test variables. An independent t test was calculated by analyzing means from two independent continuous variables. For STH risk measurements, odds ratio (OR) and 95% confidence interval (CI) were analyzed by univariate logistic regression analysis. All variables with P-value less than 0.25 in univariate analysis were accepted for further multivariate (backward stepwise) logistic regression [28]. Model fitness was determined by the Hosmer–Lemeshow statistic. A P-value of less than 0.05 (P < 0.05) was taken as significant.

## Results

### Demographic profiles and characteristics of the study population

A total of 416 Negritos (149 IJV and 267 RPS) participated in this study with ages ranging from 2 to 64 years old. By age groups, the majority of the respondents (47.1%) were primary school children aged between 7 and 12 years old. The gender ratio was 1:1. Table 2 shows the demographic profiles, socio-economic, environmental conditions and behavior characteristics in our general studied participants and according to the categories (IJV versus RPS).

**Table 2:** General demographic profiles, socioeconomic, environmental conditions and behavior characteristics of Negrito participants based on community categories.

	Variables	IJV (N = 149)	RPS (N = 267)	Overall (N = 416)
General demographic profiles		n (%)	n (%)	n (%)
Gender	Male Female	83 (55.7) 66 (44.3)	125 (46.8) 142 (53.2)	208 (50.0) 208 (50.0)
Exact age (Years)	Range Median (IQR) Mean (SD)	2–63 10.0 (9.0) 13.2 (9.9)	2–64 9.0 (6.0) 11.8 (10.1)	2–64 10.0 (7.0) 12.3 (10.0)
Age groups (Years)	1–3 4–6 7–12 13–17 >18	8 (5.4) 22 (14.8) 67 (45.0) 21 (14.1) 31 (20.8)	27 (10.1) 42 (15.7) 130 (48.7) 27 (10.1) 41 (15.4)	35 (8.4) 65 (15.6) 196 (47.1) 48 (11.5) 72 (17.3)
Family members	>7 members < 7 members	93 (62.4) 56 (37.6)	150 (56.2) 117 (43.8)	243 (58.4) 173 (41.6)
**Socio-economic status & education**				
Monthly household income^a^	<USD 125 >USD 125	88 (59.1) 61 (40.9)	188 (70.4) 79 (29.6)	276 (66.3) 140 (33.7)
Occupational status	Working^b^ Housewife Students (Primary & Secondary) Young children <7 y/o Others^c^	32 (21.5) 9 (6.0) 48 (32.3) 34 (22.8) 26 (17.4)	30 (11.2) 21 (7.9) 132 (49.4) 63 (23.6) 21 (7.9)	62 (14.9) 30 (7.2) 180 (43.3) 97 (23.3) 47 (11.3)
Past education level (adult)	Informal Primary Secondary	26 (81.3) 6 (18.8) 0	34 (82.9) 5 (12.2) 2 (4.9)	60 (82.2) 11 (15.1) 2 (2.7)
Education level (children)	Not enrolled Primary Secondary	36 (30.8) 47 (40.2) 9 (7.7)	34 (15.0) 119 (52.7) 15 (6.6)	70 (20.4) 166 (48.4) 24 (7.0)
**Sanitation and environmental**				
Source of water supply	Untreated (river, lake, etc) Treated (government piped water)	149 (100) 0	146 (54.7) 121 (43.3)	295 (70.9) 121 (29.1)
Presence of latrine	No Yes^d^	87 (58.4) 62 (41.6)	100 (37.5) 167 (62.5)	187 (45.0) 229 (55.0)
**Hygiene behaviors**				
Open/ Indiscriminate defecation	Yes^e^ No	124 (83.2) 25 (16.8)	121 (45.3) 146 (54.7)	245 (58.9) 171 (41.1)
Close contact with domestic animals	No Yes^f^	38 (25.9) 109 (74.1)	68 (25.5) 199 (74.5)	106 (25.6) 308 (74.4)
Wear shoes/slipper while outside	No Yes	109 (73.2) 40 (26.8)	107 (40.1) 160 (59.9)	216 (51.9) 200 (48.1)
Wash hands after defecation	always sometimes	42 (28.2) 107 (71.8)	135 (50.6) 132 (49.4)	177 (43.0) 239 (57.0)
Wash hands before eating	always sometimes	90 (60.4) 59 (39.6)	212 (79.4) 55 (20.6)	302 (72.6) 114 (27.4)
Boil water before drinking	No Yes	40 (26.8) 109 (73.2)	38 (14.2) 229 (85.8)	78 (18.8) 338 (81.2)
**Anti-helminthic treatment (AHT)**				
History of receiving AHT	No^g^ Yes	75 (50.3) 74 (49.7)	67 (25.1) 200 (74.9)	142 (34.1) 274 (65.9)

^a^ Cut off value of 125 USD was used because more than 60% of participants were from family with gross monthly household income of <USD 125

^b^ Providing meal to the family, mainly as a hunter gatherer, farmer (subsistence cropping), rubber tapper, boat driver, etc.;

^c^ Either not working (usually adolescent) or children who were not enrolled to school;

^d^ pour or flush toilet, pit latrine;

^e^ prefer to defecate anywhere/open (common sites: bushes, near the river) without any specific location;

^f^ own any domestic animals or always playing with them (dogs and cats are the most common);

^g^ Never received or took any anti-helminthic drug either been prescribed by a medical personnel or during mass deworming program.

With regards to the IJV Negritos, even though their locations are far from the township areas, they had significantly better socio-economic status (40.9% with a household income >USD 125) compared with those living in the RPS community (29.6%). However, about 20.4% children were not enrolled in school with only some parents sending their young children to the provided boarding school for OA children due to distance factor. In terms of hygiene behavior, walking barefoot was common and they also preferred to defecate indiscriminately (open defecation without any specific locations or at any designated latrines) mainly at the river and bushes. Moreover, about 50.3% had previously never taken or been prescribed with any anti-helminthic treatment (AHT).

Despite better development in the RPS community, the majority of the houses did not have electricity supply due to their inability to settle outstanding bills. Similar situations were seen with their piped water supplies. As a consequence, rivers (54.7%) remained the main source of water supply, especially for bathing and washing. A single latrine was commonly available in the RPS community, but the facility was not fully utilized because most of them still preferred to defecate indiscriminately as practiced in IJV. Education attainment was better in RPS, with most children being enrolled in school. However, not many of their adolescents continued or completed secondary school. Regarding drug treatment, they were highly exposed, with 74.9% having taken at least one dosage of AHT due to easier access to health-care facilities and periodic visits by medical personnel.

### Prevalence of IPIs, STH intensity, polyparasitism and diversity

Of the 416 participants, the overall prevalence of IPIs was 87.0%, with 358 (86.1%) found to be infected with at least a single species of STH and 106 (25.5%) with at least one species of intestinal protozoa (Table 3). By community categories, the prevalence of STH infections was found to be significantly higher in the IJV community compared to RPS (P = 0.02). The most dominant STH species was *T*. *trichiura* (IJV: 72.5%; RPS: 71.9%*)*, followed by *A*. *lumbricoides* (IJV: 40.3%; RPS: 44.9%) and hookworm (IJV: 26.2%; RPS: 18.7%). The prevalence of polyparasitism (having more than one type of infection) was slightly higher in the IJV community than in the RPS community but not significant. The percentage of participants positive with *Entamoeba* sp., *Blastocystis* sp. and other types of parasites (flukes, tapeworms, unidentified eggs and cysts) was greater in IJV (P = 0.01) indicating higher diversity of parasitic infections among the IJV community.

**Table 3:** Overall prevalence of IPIs and pattern of infections among IJV and RPS communities.

Intestinal parasitic infection	Overall (N = 416)		IJV (N = 149)	RPS (N = 267)	
	n (%)	95% CI	n (%)	n (%)	P value^a^
Overall any infection	362 (87.0)	83.8, 90.3	135 (90.6)	227 (85.0)	0.10
Overall any STH infection	358 (86.1)	82.7, 89.4	136 (91.3)	222 (83.1)	0.02*
Overall any protozoa infection	106 (25.5)	21.3, 29.7	46 (30.9)	60 (22.5)	0.06
**By species (STH)**:					
*T*. *trichiura*	300 (72.1)	67.8, 76.4	108 (72.5)	192 (71.9)	0.90
*A*. *lumbricoides*	180 (43.3)	38.5, 48.4	60 (40.3)	120 (44.9)	0.36
Hookworm	89 (21.4)	17.4, 25.4	39 (26.2)	50 (18.7)	0.08
*Strongyloides* sp.	7 (1.7)	0.4, 2.9	3 (2.0)	4 (1.5)	^¥^0.71
**By species (Protozoa)**:					
*Entamoeba/Dientamoeba* sp.	91 (21.9)	17.9, 25.9	41 (27.5)	50 (18.7)	0.04*
*Blastocystis* sp.	18 (4.3)	2.4, 6.3	10 (6.7)	8 (3.0)	0.07
*Giardia* sp.	16 (3.8)	2.0, 5.7	8 (5.4)	8 (3.0)	0.23
*Cryptosporidium* sp.	12 (2.9)	1.2, 4.5	4 (2.7)	8 (3.0)	0.56
Others parasites (Flukes, Tapeworms, unidentified cyst and eggs)	37 (8.9)	6.2, 11.6	21 (14.1)	16 (6.0)	0.01*
**Monoparasitism & Polyparasitism status**					
Single infection	144 (34.6)	30.0, 39.2	48 (32.2)	96 (36.0)	0.44
Double infection	140 (33.7)	29.1, 38.2	56 (37.6)	84 (31.5)	0.21
Triple and more infection	78 (18.8)	15.0, 22.5	31 (20.8)	47 (17.6)	0.42

CI = Confidence Interval

^a^ The P values were calculated based on Pearson’s chi-square test between the two categories of community (IJV versus RPS)

^¥^ Fisher’s exact test

* Significant difference P<0.05

#### *Trichuris trichiura* infection

The prevalence of *T*. *trichiura* infection was almost the same (P = 0.90) in both communities (Table 3). By gender, the female Negritos tend to be infected with this species at an earlier age (1–3 years old) compared to males from the same age category in both IJV and RPS **(**S2 Fig). However, as age increases, the percentage of *T*. *trichiura* was comparable between males and females in IJV. In contrast, in RPS, the fraction of adult females positive with this parasite was lower compared with their younger generation but 100% positivity rate was observed among adult males (S2 Fig). However, the percentage of individuals with severe intensity of *T*. *trichiura* was significantly higher in RPS (17.2%) compared to IJV (6.5%) (P = 0.01). (Table 4). By gender, the percentage of moderate to severe infection of *T*. *trichiura* was found to be higher in females than in males in both IJV (females: 68.9%, males: 58.7%; P = 0.28) and RPS (females: 67.3%; males: 62.4%; P = 0.48) communities. By age groups, young children aged <10 years old had higher percentage of moderate to severe rate than those aged >10 years old in both communities but was statistically significant only in RPS (age < 10: 71.6%, age > 10: 55.3%; P = 0.02).

**Table 4:** Intensity of STH infections among the Negritos in Peninsular Malaysia according to STH species and comparison between communities (IJV versus RPS).

	Inland Jungle Villages (IJV)		Resettlement (RPS)			
STH species	n (%)	Mean epg (SE)	n (%)	Mean epg (SE)	P value^a^	P value^b^
***T*. *trichiura***						
Overall	N = 108	3,105.3 (291.1)	N = 192	5,453.8 (612.5)	-	0.001*
Light	40 (37.0)	768.0 (30.3)	67 (34.9)	745.9 (23.4)	0.71	0.57
Moderate	61 (56.5)	3,652.3 (244.3)	92 (47.9)	4,097.3 (255.9)	0.15	0.23
Severe	7 (6.5)	11,694.9 (382.0)	33 (17.2)	18,793.9 (2319.5)	0.01*	0.01*
***A*. *lumbricoides***						
Overall	N = 60	16,592.0 (2316.5)	N = 120	18,698.7 (1794.1)	-	0.47
Light	24 (40.0)	2,851.0 (251.3)	36 (30.0)	3,182.9 (193)	0.18	0.30
Moderate	27 (45.0)	16,739.6 (1921.7)	60 (50.0)	14,257.8 (1460.2)	0.53	0.33
Severe	9 (15.0)	52,792.0 (767.1)	24 (20.0)	53,074.3 (533.6)	0.41	0.78
**Hookworm**						
Overall	N = 39	1,963.1 (191.2)	N = 50	1,375.6 (112.9)	-	0.01*
Light	23 (59.0)	1,283.5 (81.3)	38 (76.0)	1,037.8 (69.9)	0.09	0.03*
Moderate	14 (35.9)	2,497.7 (128.8)	12 (24.0)	2,445.3 (218.7)	0.22	0.21
Severe	2 (5.1)	6,036.0 (84.0)	-		-	-

STH = Soil transmitted helminths; SE = Standard error of mean; epg = eggs per gram

^a^ The P values were obtained from the comparison of the number of infected participants for each variable (%) between IJV and the RPS communities (Pearson chi-square test).

^b^ The P values were calculated from the different of the mean epg values between IJV and the RPS communities (Independent t- test);

*Significant difference P < 0.05

#### *Ascaris lumbricoides* infection

For both prevalence (Table 3) and percentage of individuals with severe *A*. *lumbricoides* infection (Table 4), although they did not reach a statistically significant level, higher trend was observed in the RPS community [(RPS = prevalence 44.9%; severe infection 20.0% versus IJV = prevalence 40.3%; severe infection 15.0%; P = 0.36, P = 0.41)]. By gender, the females had higher prevalence of infection in both communities (IJV = females: 45.5%, RPS: 36.1%; P = 0.25, RPS = females: 47.9%, males 41.6%; P = 0.30). However, the number of males with moderate–severe infection (66.7%) was greater than the females (53.3) in IJV (P = 0.29), but comparable in the RPS community (males: 73.1%, females: 72.1%; P = 0.90). By age groups, the prevalence of *A*. *lumbricoides* infection was higher among the younger population and lower among the adults in both communities (S2 Fig). With regard to intensity, 65.6% infected children aged <10 years in the IJV community had moderate to severe infection compared with 53.6% with the same intensity level among older individuals (i.e., >10 years; P = 0.34). However, in the RPS, not much difference was observed within age groups in terms of severity (P = 0.81).

#### Hookworm *infection*

Both prevalence (Table 3) and individuals with moderate to severe hookworm infections (Table 4) were higher in the IJV community (26.2%, 41.0%) compared to RPS (18.7%, 24.0%) (P values = 0.08, 0.09). Unlike infections by *T*. *trichiura* and *A*. *lumbricoides*, hookworm infection was higher in males in both communities. However, the severity of hookworm was found to be greater (moderate–heavy) in females than in males as observed both in IJV (female: 53.8%, male: 30.8%; P = 0.16) and RPS (female: 35.0%, male: 23.3%; P = 0.37). By age group, the hookworm prevalence rate was maintained across all age groups, including adults in both types of communities. However, the intensity was found to be higher among older participants than among young infected persons (IJV = age <10: 27.8%, age>10: 47.6%; P = 0.20; RPS = age <10: 23.1%, age>10: 33.3%; P = 0.42).

### Risk factors of STH infections

Our findings showed that Negritos who live in IJV had 2.1 times greater risks (95% CI: 1.1, 4.1; P = 0.02) to be infected with STH than RPS. We then compared the associations between STH infections with demographic, socio-economic and environmental circumstances, and personal hygiene behavior variables (Table 5). In the IJV community, univariate analysis indicated significant association between children aged <12 years old (P = 0.03), persons who defecated indiscriminately (commonly at the bushes) (P = 0.04), walking barefoot (P = 0.003) and had close contact with domestic animals (P = 0.002) with the presence of STH. Meanwhile in RPS, the Negritos with low household monthly income (<USD 125) had 3.9 times greater risks (P <0.001) to be infected with STH. Other significant risk factors in RPS communities were related to poor sewage disposal system (P = 0.01) and bad hygiene behaviors, such as not washing hands after playing or contact with soil (P<0.001), not washing fruits and vegetables before eating (P = 0.03) and closer contact with animals (P< 0.001).

**Table 5:** Potential risk factors associated with STH infections among IJV and RPS communities (logistic regression univariate analysis).

	Total infected participant (n = 358)					
Variables	Inland jungle villages, IJV (N = 149)			Resettlement, RPS (N = 267)		
	% infected	OR (95% CI)	P value	% infected	OR (95% CI)	P value
**Gender** Female Male	93.9 89.2	1.9 (0.6, 6.4) 1	0.31	86.6 79.2	1.7 (0.9, 3.3) 1	0.11
**Age** *<12* *>12*	95.5 85.2	3.6 (1.0, 12.4) 1	0.03*	84.5 81.9	1.2 (0.6, 2.3) 1	0.73
**Family size** >7 members <7 members	90.3 92.9	1 1.4 (0.4, 4.8)	0.60	86.0 79.5	1.6 (0.8, 3.0) 1	0.16
**Household monthly income** <USD 125 >USD 125	94.3 86.9	2.5 (0.8, 8.1) 1	0.11	89.4 68.4	3.9 (2.0, 7.5) 1	<0.001*
**Category occupation** Working Not working	87.5 92.3	1 1.7 (0.5, 6.0)	0.48	89.7 82.4	1.8 (0.5, 6.4) 1	0.44
**Source of water supply** Untreated (river, lakes, etc.) Treated (Government piped water)	NA	NA	NA	85.6 80.2	1.5 (0.8, 2.8) 1	0.24
**Open/Indiscriminate defecation** Yes (open areas, bushes, etc.) No (at latrine)	93.5 80.0	3.6 (1.0, 12.2) 1	0.04*	86.8 80.1	1.6 (0.8, 3.2) 1	0.15
**Sewage disposal** Outdoor (improper) Proper drainage	NA	NA	NA	88.9 76.4	2.5 (1.3, 4.8) 1	0.01*
**Washing hands before eating** No Yes	91.4 91.2	1.0 (0.3, 3.2) 1	0.97	84.3 82.2	1.2 (0.6, 2.2) 1	0.64
**Washing hands after defecation** No Yes	92.5 88.4	1.6 (0.5, 5.2) 1	0.52	85.2 81.3	1.3 (0.7, 2.5) 1	0.42
**Washing hands after playing/contact with soil** No Yes	93.5 85.4	2.4 (0.8, 7.7) 1	0.12	91.1 75.0	3.4 (1.7, 6.9) 1	<0.001*
**Wearing shoes /slipper outside of house** No Yes	95.4 80.0	3.6 (1.1, 11.6) 1	0.003*	88.8 79.4	2.1 (1.0, 4.2) 1	0.04*
**Washing fruits/vegetables before eating** No Yes	94.9 88.9	2.3 (0.6, 8.9) 1	0.20	92.7 80.7	3.1 (1.0, 8.9) 1	0.03*
**Boiling water before drinking** No Yes	95.0 89.9	2.1 (0.5, 10.1) 1	0.33	92.1 81.7	2.6 (0.8, 8.9) 1	0.11
**Close contact with domestic animals** Yes No	95.5 79.5	3.8 (1.2, 12.1)	0.002*	92.6 65.2	6.6(3.3,13.5)	<0.001*

NA: Not applicable; Reference group = Odds ratio, OR (1); CI: Confidence Interval, P < 0.05 indicated significant association

Table 6 shows the result of further multivariate models for final significant predictors for STH infections in both the IJV and RPS communities. For the IJV community, only three factors; closer contact with animals, indiscriminate defecation, and walking barefooted (P values: 0.01, 0.03 and 0.04, accordingly) which were all grouped under poor hygiene behaviors, remained as the significant predictors. Goodness-of-fit by the Hosmer–Lemeshow test indicated the model fits the data well (*χ*^2^ = 2.20; df = 4, P = 0.69). With regards to RPS community, from eleven variables, five factors were retained; low monthly income of less than USD 125 (P < 0.001), improper sewage disposal (P = 0.03), poor hygiene behavior of not washing hands after playing with soil (P = 0.01), indiscriminate defection (0.04) and those with a closer contact with animals (or have domestic animals in their household) (P < 0.001) had a higher prevalence rate of STH than their counterparts with STH infections (Hosmer–Lemeshow test: *χ*^2^ = 5.83, df = 7; P = 0.56).

**Table 6:** Potential risk factors associated with STH infections among IJV and RPS communities (multivariate logistic regression model).

Variables	IJV	P value	RPS	P value
	AOR (95% CI)		AOR (95% CI)	
**Factors- General demographic**				
Gender (females)	-	-	1.6 (0.8, 3.6)	0.20
Age (<12 years old)	1.7 (0.3, 9.6)	0.60	-	-
Family size (>7 members)	-	-	1.9 (0.8, 4.2)	0.10
**Factors-socioeconomic and environmental**				
Household monthly income (<USD 125)	2.1 (0.6, 7.6)	0.27	3.5 (1.6, 7.5)	0.001*
Source of water supply (untreated)	-	-	1.5 (0.6, 3.8)	0.39
Sewage disposal (outdoor, improper)	-	-	4.7 (1.2, 18.8)	0.03*
**Factors- personal hygiene behaviors**				
Open/ Indiscriminate defecation (yes)	5.0 (1.2, 21.3)	0.03*	4.5 (1.1,18.6)	0.04*
Washing hands after playing/contact with soil (no)	1.2 (0.3, 5.5)	0.80	2.8 (1.2, 6.2)	0.01*
Wearing shoes/slipper when outside (no)	3.6 (1.0, 12.7)	0.04*	1.4 (0.6, 3.6)	0.40
Washing fruits/vegetables before eating (no)	2.0 (0.4, 9.9)	0.40	1.4 (0.4, 5.7)	0.59
Boiling water before drinking (no)	-	-	1.4 (0.3, 6.9)	0.67
Closer contact with domestic animals (yes)	6.9 (1.7, 27.1)	0.01*	5.7 (2.7, 12.4)	<0.001*

AOR: Adjusted odds ratio; CI: Confidence interval

*Significant predictor of STH infection (P< 0.05)

## Discussion

This study attempts to compare the current status of intestinal parasitism between two types of Negrito communities which have different environmental and ecological settings, separated due to demarginalization and modernity. Results revealed higher IPIs prevalence and a significantly greater proportion of STH prevalence among those staying in inland forest, IJV (>91%), compared to those staying in resettled RPS. The prevalence of IJV Negritos harboring other different species of intestinal parasites, such as *Entamoeba* sp., *Blastocystis* sp., flukes and tapeworm was also higher, indicating higher diversity of parasitic infections compared to the RPS Negritos. This finding in IJV is in agreement with the earliest finding by Dunn in 1972, which indicated high intestinal parasitism with > 96% STH prevalence rate among a group of Negritos who had not undergone resettlement [21]. It could be probably related to their vast exposure to more varied ecosystem of flora and fauna due to the remote rainforest setting. According to Dunn, the prevalence and diversity of parasitic species in the human population is related to their environment and the complexity of the surrounding ecosystem [21, 29]. For example, the diversity of parasites in African and Malaysian hunter-gatherers living in a tropical rain forest was reported to be two times higher than bushman and Australian aborigines who live in a species-poor environment [29]. Thus, despite *in situ* improvement by provision of basic infrastructure, the contemporary IJV Negritos are still largely involved in hunting, fishing and forest produce gathering. These factors may predispose them to higher susceptibility to a greater variety of parasites, hence, the higher prevalence rate and higher diversity of parasitic species in the IJV community. When the new habitat and environmental setting is drastically changed and no longer within the forested area as observed in RPS community, some parasite species that may require intermediate hosts are gradually eliminated, reducing the prevalence and diversity of the species in this community [10, 21].

With regards to STH species, both communities had the same pattern of parasite predominance, where *T*. *trichiura* is the most dominant species, with a comparable prevalence rate (>70%) in both IJV and RPS. Recent studies conducted on the other larger tribes, the Senoi and Proto-Malay, also reported of *T*. *trichiura* predominance, with prevalence ranging from 35.0% to 77.0% [12, 14, 16, 17]. Based on this current situation, *T*. *trichiura* could be the main intestinal helminth responsible for the current STH epidemiological distribution among OA across Peninsular Malaysia (regardless of locations in the remote forest, or at the peripheries of towns), which is contrary to findings in other countries that recorded *A*. *lumbricoides* predominance [30–32]. High resistance towards anti-helminthic (ATH) interventions could be the most possible reason [33]. As revealed in previous findings, the cure rate for *T*. *trichiura* following treatment with a single dosage of albendazole or mebendazole were 28% and 36%, respectively [34]. There is yet a clear explanation on this local problem; however, *T*. *trichiura* resistance against albendazole/mebendazole (these drugs are used widely in Malaysia for STH treatment) could be related to high frequency or inappropriate use of drugs [35] and poor adherence or low compliance following periodic AHT treatment [36].

Although the overall prevalence of STH infection was lower in the RPS Negritos, this community has the highest helminth burden of *T*. *trichiura*. The latter finding is opposed to our pre-assumption hypothesis of STH reduction in the RPS community due to the fact that they had better access to health-care facilities, which supposedly increase the success of controlling the STH burden. Nevertheless, our findings support the previous ecological theory which indicated that any changes in the environment setting, could lead to differential exposure to infection [21, 37]. This means that once a population in the jungle is relocated to simplified RPS resettlement, the prevalence of certain parasitic species is reduced, but other parasites, which require neither intermediate host nor complex lifecycle would arise with higher intensity. In the case of RPS Negritos, although they have moved into the peripheries, the situation was further aggravated by the adoption of similar poor hygienic behaviors and the practice of open defecation which could lead to high degree of soil contamination.

From our observation, the soil around the houses is the most profoundly contaminated with parasitic eggs because young children tend to defecate on the ground around the houses or at least within the RPS vicinity. Therefore, the *T*. *trichiura* infection which can be acquired simply by fecal- oral route, could be found in higher intensity within the context of simplified overcrowded RPS due to limited land availability (e.g., wedged between the road and other Malay communities) and larger population size (between 170–450 people) [38]. Moreover, their more sedentary lifestyles could predispose them with frequent contacts with contaminated agents such as soil, food, water [39]. This factor was supported by our finding which indicated higher infection with *T*. *trichiura* and *A*. *lumbricoides* particularly among adult females. They have higher exposure to the infections due to lower mobility, spending more time at inhabitant areas and are mainly responsible for taking care of the young children who defecate indiscriminately/open and playing in their housing areas. Within a sedentary population, the patterns of transmission are spatially and temporally stable, which may promote higher intensity by continual reinfection of parasites between group members [38]. The new alteration of the host’s lifestyles in the simplified RPS environment, may also contribute to some ecological displacement probably by competition for resources among the co-existing parasites [29, 40, 41]. Similar finding of higher STH intensity due to high soil contamination was also reported among Amerindian population in Brazil who have undergone resettlement [42]. Unfortunately, the comparison on the rate of transmission and reinfection status after deworming was not examined in this study; hence it is a limitation factor that needs to be addressed in future studies. Nonetheless, previous study among OA children in 2008 reported high re-infection rate with almost half of the participants being re-infected again with STH after 3 months post treatment [43]

In IJV community, despite higher prevalence, the lower burden of *T*. *trichiura* were possible because of their ample inhabitant areas with low population density (between 50 and 300 people), hence reducing the load of contaminated soil. It is believed that the contamination is well dispersed and scattered, which reduces rapid reinfection and transmission rate. High mobility rate and physical activities among the IJV people compared to the RPS Negritos would probably reduce the burden of the parasite. Even though the IJV people are no longer nomad, they still traditionally move around for foraging and gathering of forest resources within their territory, which prevented them from becoming sedentary. Although exposed to higher and more diverse parasitic infections, the environmental setting of IJV community reflects their adaptation and the concept of living in harmony with their natural forest ecosystems and allowing nature to rehabilitate hence reducing the burden of predominant STH.

In addition, there is another postulation focusing on the role of the parasitic infections as a natural controller of population regulation and agent of natural selection among indigenous jungle community [29]. Any disturbance of the environment may induce destabilization of the diseases by either disappearing or becoming more severe on certain individuals [29,44]. The theory was supported by the nature of the parasites, with most of them coexisting, either without causing significant harm or with a wide range of morbidity effects among the indigenous [29, 45]. Severe morbidity and mortality can only be caused by factors such as a severe load of worms, malnutrition and low immune system of the host [45]. This aspect, perhaps, should be addressed and is vital for further exploration in the future.

In this study, not much difference was discovered in the prevalence and the intensity of the *A*. *lumbricoides* infection in both IJV and RPS communities. Because of the similar fecal-oral route of transmission, most of the infected individuals were also harboring the *T*. *trichiura* parasite. The co-infection of these two parasites were common and have been reported among OA in Malaysia [43].

For hookworm infection, even though the prevalence and intensity of hookworm infection was found to be higher in the IJV people compared to the RPS, the finding was not significant. While most of the cases were mild in both communities, the number of individuals with moderate to severe infections were found to be higher in the IJV community. The most probable reason is because, the IJV Negritos often walked barefooted and this predisposed them to larva hookworm penetration via skin. Prior to the 1980s, when OA development was still in an early phase, hookworm infection was almost universal in nomadic Negritos and the leading cause of STH infection [10, 21] with prevalence rate up to 95.0% [21, 46]. However, the rate of hookworm infection has reduced to less than 35.0% among the OA communities since the 1990s, owing primarily to the vigorous implementation of mass deworming program [10, 47, 48]. The success of the control program was also heightened with the acknowledgment of the importance of wearing shoes outdoors as now practiced commonly among the OA in RPS community.

We report the risk factors of STH and compared the findings between each community. In RPS, low socio-economic status and high poverty rate served as one of the significant predictors for STH acquisition. Despite its commendable objective, development in the RPS communities has been slow even though they have been relocated for a decade [6]. The number of housing units was also limited and was built with poor quality materials and is unsatisfactory. In reality, some resettlement projects were results of forced migration due to logging projects, construction of large-scale dams and other infrastructure, land development schemes and plantations which largely benefited non-tribal minorities [49]. It has been reported that very few communities have improved, while most of the OA communities following demarginalization have become poorer [7]. This could be due to unfavorable locations of the new resettlements, which lack traditional resources of living out of forest products and poor social adaptation to new and modern environment [7]. Under these conditions and surroundings, the RPS Negritos are unable to practice hunting and forest gathering even though these activities are symbolically significant to their identity and self-characterization [50]. Low income leads to difficulty in adapting to the new simplified situation, increasing poverty and dependency on the government which lead to higher burden of STH infection [40].

Human factor such as poor hygiene behavior (e.g. not washing hands after playing/contact with soil) also heightened the risk of getting STH infections in RPS community. The practice of open/indiscriminate defecation worsened the situation. Despite housing infrastructure provided by the government, most of the RPS Negritos refused to use the toilet for defecation due to cultural belief that defecation is not suitable to be carried out in the house [51]. The condition of the subsidized toilets and latrines which were not properly built and without sufficient piped water facilities or proper maintenance were also part of the reasons why there are poor uptake by this community. As a result, the toilets were often used as storage rooms [52]. These factors contributed to frequent environmental contamination and consequently massive accumulation of parasitic eggs in their soil environment leading to the endless problem of *T*. *trichiura* and *A*. *lumbricoides* infections in this RPS community.

In contrast, in the IJV community, socio-economic factors were not associated with intestinal parasitism because all had equal risks of infection due to their homogeneous lifestyle. Previous studies even suggested that the *in situ* development of the IJV have improved the livelihood probably due to the fact that they were not relocated to the new resettlement areas [7]. In addition, their involvement in eco-tourism industry such as a nature guide in the forest has helped their socio-economic conditions. As their habitat were in the forest, the hunting and gathering life styles were still highly relevant, thus providing better continuity in their life [7]. However, the risk factors associated with STH among them were mainly related to poor hygienic behavior, such as closer contact or having domestic animals in household, open/indiscriminate defecation (around the houses, at the bushes or near the streams) and walking barefoot. Walking barefoot outdoors most likely served as a significant risk factor for higher hookworm infection in the IJV community because hookworm infection is acquired through larvae skin penetration.

Individuals who have close contact with domestic animals such as dog and cat are highly predisposed to STH infections in both the IJV and RPS communities. These animals might be part of the mechanical transmission of human and animal STH because promiscuous and open/indiscriminate defecation lifestyles are obvious in both IJV and RPS communities. The role of domestic animals in transmitting STH has been acknowledged in previous studies [12, 51, 53, 54]. Animals also can act as a biological transmitter, reservoir and environmental contaminators for certain STH, such as hookworm [54] and *A*. *lumbricoides* infections [55] in populations where open discrimination is usual. It is also important to note that these animals can be a source of zoonotic helminths since a wide range of parasites such as *Toxocara* sp., *Ancylostosma ceylanicum* and *Ancylostoma caninum* have been reported in the fecal samples of dogs and cats [54, 56].

**Fig 1:**
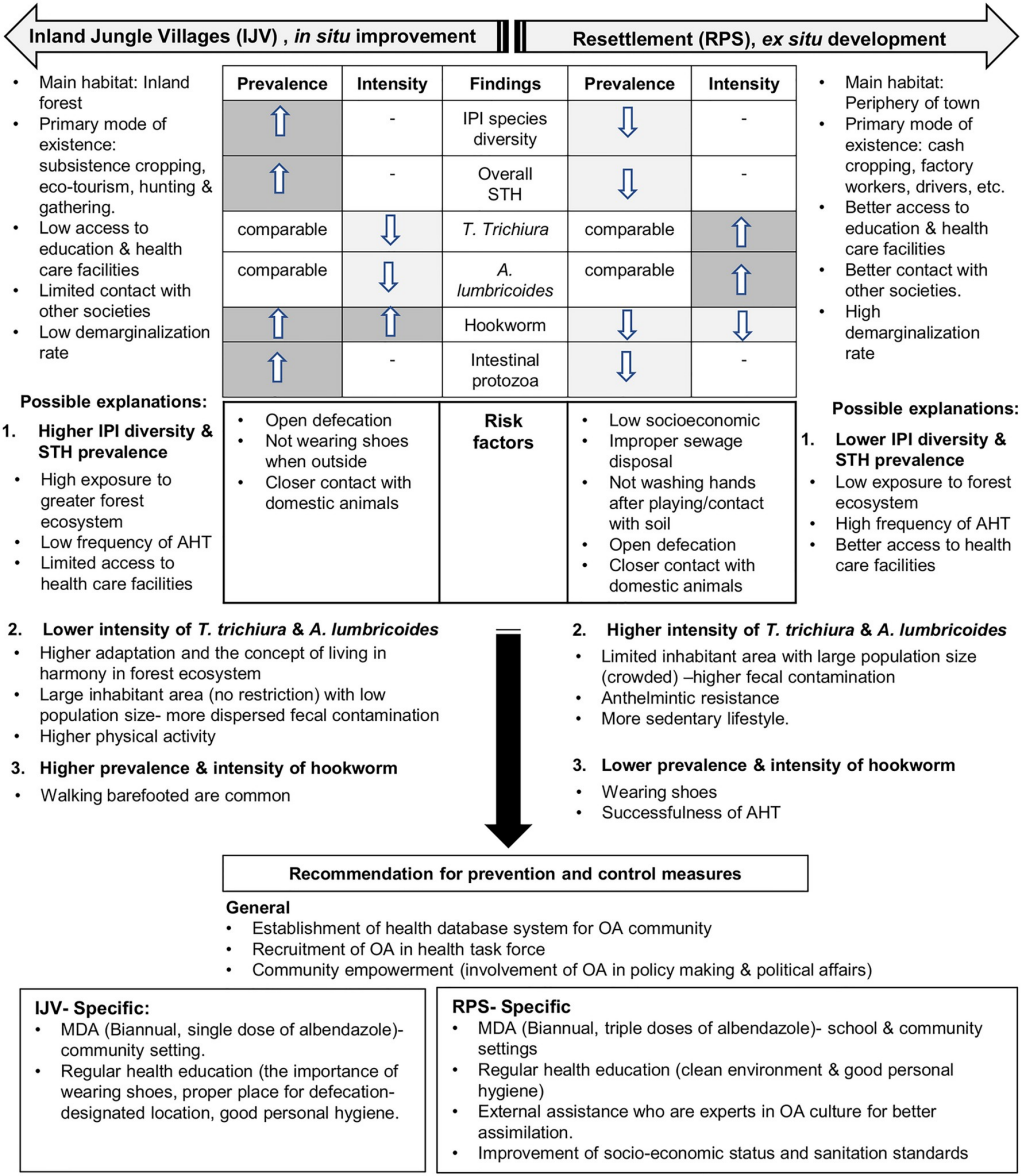
A conceptual summary explaining the pattern of intestinal parasitism by comparing IJV and RPS Negrito communities.

Therefore, different strategies of STH control and prevention are needed and should be extended to all indigenous OA populations, according to their habitat, environmental and ecological settings **(**Fig 1). Instead of targeting IPIs and STH prevalence, priority should be given to helminth-burden reduction. This finding needs to be addressed effectively because moderate and severe intensities of these infections are known to cause malnutrition, anemia, poor cognitive functions and learning ability. The consequences of these effects, especially among the young RPS OA generation, may affect their adulthood productivity and cause their communities to plunge into further poverty and low quality of life. In IJV community, by addressing the fact that they are no longer nomadic, the current trend of *T*. *trichiura* predominance and further demarginalization gives some predictions that their situation will be similar to what happened in the RPS, if effective measures are not taken seriously. Apart from low socio- economic and poor sanitation system, other main factors of STH persistency among OA communities were thought to be due to the failure in delivering effective health education, including lacking sensitivity towards their culture among the health personnel, communications barrier and the use of ineffective materials [57]. Proper implementation with huge participation by the OA themselves is crucially needed. The importance of WASH (water, sanitation and hygiene) improvement [58] including the importance of proper defecation should be emphasized in both RPS and IJV communities. This approach would also be feasible in controlling the transmission from domestic animals, as observed in this study.

For effective results, the influence of the head man or ‘Tok Batin’ is important in health education because the information could be delivered efficiently in their own language. High recruitment and involvement of more OA people in the health-care taskforce would help to alleviate and lessen their worries on the health issue, consequently better participation and uptake among them as seen in the Australian indigenous [59]. In the long run, more education, training and employment in health-related fields should be carried out among the OA. Apart from that, indigenous cultures and behavior studies should be introduced and included to educate and train health workers to heighten sensitivity and understanding of OA health problems [60, 61]. For mass drug administration, we suggest biannual triple dose regimens of albendazole in this RPS community for higher efficacy rate [62], taking into consideration higher worm burden and *T*. *trichiura* resistance towards AHT. Meanwhile, in the IJV communities, a biannual single dose of albendazole may be suffice in this community due to lower intensity and better adaptation among them. In addition, a proper database system solely for the OA community containing all records pertaining to AHT are strongly needed for easier monitoring and documentation. Apart from that, this database system may help to increase the compliance rate of treatment and possibly reduce the drug inefficiency and resistance as documented previously in *T*. *trichiura* infection [35].

### Limitations

There are a few limitations that need to be considered while interpreting the present findings. Firstly, the findings were based on one time point collection within a certain period without being able to identify the relationship of current infection with previous and future infections. Secondly, the study population was quite small and represented only 8.3% of the total Negrito population. Therefore, it may not reflect the community of OA as a whole. Thirdly, the sampling was based on convenient, snowball method and this may provide some biased outcomes, as the sampling is not random. Nevertheless, the sampling approach was the most appropriate and feasible considering the challenges and strict procedures to recruit the OA participants, especially in the IJV communities. In addition, the prevalence of *S*. *stercoralis* could be underestimated because all the methods used have a low sensitivity for this particular parasite.

### Conclusions

Despite a slight reduction of intestinal parasitism in the RPS, the *ex situ* development plan has not profoundly contributed to a positive impact on the status of helminth infestation among the OA. In fact, the burden of *T*. *trichiura* was more intense due to further poverty, adoption of similar poor hygienic behaviors and lack of proper sanitation. Nevertheless, the concept of RPS for demarginalization and consequently reduction of STH endemicity, can still be further improved taking into consideration holistic development of proper sanitation and socio-economic improvement, biannual mass deworming programs, and more importantly coupled with health educations strategies to change mindsets in both RPS and IJV communities. For long-term intervention and better uptake, these strategies must inclusive, with the participation and empowerment of the OA of the respective community, respecting their traditional customs and accompanied by recruitment of more OA people in the health-care taskforce. Importantly, the increased involvement of OA people in policy-making and political affairs which are related to the changes of ecological, social and economic drivers among them may indirectly improve their health and consequently reduce the burden of helminth infections. Otherwise, the malady among them will remain unchanged and unsolved, increasing the cost of economic burden in controlling these infections.

## Supporting information

S1 Checklist STROBE checklist. (DOCX)

S1 Fig The sampling locations and the two categories of Negrito (indigenous) communities. (A) A map showing the sampling locations which involved all sub-tribes of the Negrito. The Negritos are concentrated mainly in the northern states of Peninsular Malaysia. (B) The Inland Jungle Villages (IJV)—*In situ* improvement. (C) The Resettlement (RPS)—*Ex situ* development. *Source*: *Map was recreated from a blank map of Peninsular Malaysia available at* https://publicdomainvectors.org/en/free-clipart/Blank-map-of-peninsular-Malaysia/50795.html.
(TIF)

S2 Fig Prevalence of STH by each species according to age groups and gender (IJV versus RPS). The females tend to be infected by STH at an early age of life compared to the male Negritos in both communities. In RPS, 100% positivity rate of *T*. *trichiura* infection was observed among the adult males, most likely because the targeted group for AHT treatment previously was pre- and school aged children. The rate of hookworm infection was higher in adult males and increased with age.
(TIF)
