# Supplementary material for: Prevalence, intensity and associated risk factors of soil transmitted helminth infections: A comparison between Negritos (indigenous) in inland jungle and those in resettlement at town peripheries
Source: PLoS Negl Trop Dis. 2019 Apr 22;13(4):e0007331. doi: 10.1371/journal.pntd.0007331 (PMC6497322; doi:10.1371/journal.pntd.0007331)
Supplement: S1 Checklist — (DOCX) [file pntd.0007331.s001.docx]

STROBE Statement—Checklist of items that should be included in reports of ***cross-sectional studies***

|  | Item No | Recommendation |
| --- | --- | --- |
| **Title and abstract** | 1 | (*a*) Indicate the study’s design with a commonly used term in the title or the abstract **[Page 2 : Abstract; Lines 29-33]** |
|  |  | (*b*) Provide in the abstract an informative and balanced summary of what was done and what was found **[ Page 2: Abstract; Lines 30-44]** |
| Introduction | | |
| Background/rationale | 2 | Explain the scientific background and rationale for the investigation being reported  **[Page 3: Author summary: Lines 53-69, Pages 4-5; Introduction: Paragraph 1-4, Lines 111-120]** |
| Objectives | 3 | State specific objectives, including any prespecified hypotheses **[Page 5; Introduction: Paragraph 3 & 5, Lines 118-120, Lines 129-132]** |
| Methods | | |
| Study design | 4 | Present key elements of study design early in the paper **[Page 6; Study design, sample size calculation and study population: Paragraph 1 & 2, Lines 142-158]** |
| Setting | 5 | Describe the setting, locations, and relevant dates, including periods of recruitment, exposure, follow-up, and data collection **[ Page 6: Study design, sample size calculation and study population; Paragraph 2, Lines 155-171]** |
| Participants | 6 | (*a*) Give the eligibility criteria, and the sources and methods of selection of participants **[Pages 6: Study design, sample size calculation and study population; Lines 145-148, 155-160, Page 8: Stool sample collection, Lines 213-216].** |
| Variables | 7 | Clearly define all outcomes, exposures, predictors, potential confounders, and effect modifiers. Give diagnostic criteria, if applicable [**Page 6 & 7: Study design, sample size calculation and study population & Questionnaire; Lines 155-160, 238-239, 242-253)** |
| Data sources/ measurement | 8* | For each variable of interest, give sources of data and details of methods of assessment (measurement). Describe comparability of assessment methods if there is more than one group **[Study design, sample size calculation and study population, paragraph 2, Line 155-158; Questionnaire, paragraph 1, Lines 197-210]** |
| Bias | 9 | Describe any efforts to address potential sources of bias **[Page 23; Limitation Lines 591-600 ]** |
| Study size | 10 | Explain how the study size was arrived at **[Page 7: Study design, sample size calculation and study population, Paragraph 3, Lines 172-178]** |
| Quantitative variables | 11 | Explain how quantitative variables were handled in the analyses. If applicable, describe which groupings were chosen and why **[Page 9: Data management and statistical analysis; Paragraph 1, Lines 242-245]** |
| Statistical methods | 12 | (*a*) Describe all statistical methods, including those used to control for confounding  **[Pages 9&10: Data management and statistical analysis; Paragraph 1 &2 , Lines 242-263]** |
|  |  | (*b*) Describe any methods used to examine subgroups and interactions **[Pages 9&10: Data management and statistical analysis; Paragraph 1 &2 , Lines 242-263]** |
|  |  | (*c*) Explain how missing data were addressed **[N/A ]** |
|  |  | (*d*) If applicable, describe analytical methods taking account of sampling strategy  **[ N/A]** |
|  |  | (*e*) Describe any sensitivity analyses **[ N/A]** |
| Results | | |
| Participants | 13* | (a) Report numbers of individuals at each stage of study—eg numbers potentially eligible, examined for eligibility, confirmed eligible, included in the study, completing follow-up, and analysed **[ Page 7: Materials & methods, page 7, Lines 179-183; Page 10-Results paragraph 1]** |
|  |  | (b) Give reasons for non-participation at each stage **[Page 7: Materials & methods, page 7, Lines 179-183]** |
|  |  | (c) Consider use of a flow diagram **[ N/A]** |
| Descriptive data | 14* | (a) Give characteristics of study participants (eg demographic, clinical, social) and information on exposures and potential confounders **[Results: Demographic profiles and characteristics, Paragraphs 1-3, Lines 267-290]** |
|  |  | (b) Indicate number of participants with missing data for each variable of interest  **[ N/A]** |
| Outcome data | 15* | Report numbers of outcome events or summary measures **[ Results: Demographic profiles and characteristics; Paragraphs 2-3, Prevalence-Paragraph 1-4 and Risk factors-Paragraphs 1 & 2)** |
| Main results | 16 | (*a*) Give unadjusted estimates and, if applicable, confounder-adjusted estimates and their precision (eg, 95% confidence interval). Make clear which confounders were adjusted for and why they were included **[Results: Table 2- Table 6 ]** |
|  |  | (*b*) Report category boundaries when continuous variables were categorized  **[N/A]** |
|  |  | (*c*) If relevant, consider translating estimates of relative risk into absolute risk for a meaningful time period **[ N/A]** |
| Other analyses | 17 | Report other analyses done—eg analyses of subgroups and interactions, and sensitivity analyses **[ N/A ]** |
| Discussion | | |
| Key results | 18 | Summarise key results with reference to study objectives **[ Discussion: Paragraphs 1-3, paragraphs 7-8]** |
| Limitations | 19 | Discuss limitations of the study, taking into account sources of potential bias or imprecision. Discuss both direction and magnitude of any potential bias **[Limitation, paragraph 1; Lines 591-600]** |
| Interpretation | 20 | Give a cautious overall interpretation of results considering objectives, limitations, multiplicity of analyses, results from similar studies, and other relevant evidence  **[Discussion: Paragraph 1-8]** |
| Generalisability | 21 | Discuss the generalisability (external validity) of the study results **[Discussion and conclusion: Paragraph 9-13]** |
| Other information | | |
| Funding | 22 | Give the source of funding and the role of the funders for the present study and, if applicable, for the original study on which the present article is based **[Included in the submissions]** |

*Give information separately for exposed and unexposed groups.

**Note:** An Explanation and Elaboration article discusses each checklist item and gives methodological background and published examples of transparent reporting. The STROBE checklist is best used in conjunction with this article (freely available on the Web sites of PLoS Medicine at http://www.plosmedicine.org/, Annals of Internal Medicine at http://www.annals.org/, and Epidemiology at http://www.epidem.com/). Information on the STROBE Initiative is available at [www.strobe-statement.org](http://www.strobe-statement.org).
