# Supplementary figures and images for: Prevalence, intensity and associated risk factors of soil transmitted helminth infections: A comparison between Negritos (indigenous) in inland jungle and those in resettlement at town peripheries
Source: PLoS Negl Trop Dis. 2019 Apr 22;13(4):e0007331. doi: 10.1371/journal.pntd.0007331 (PMC6497322; doi:10.1371/journal.pntd.0007331)

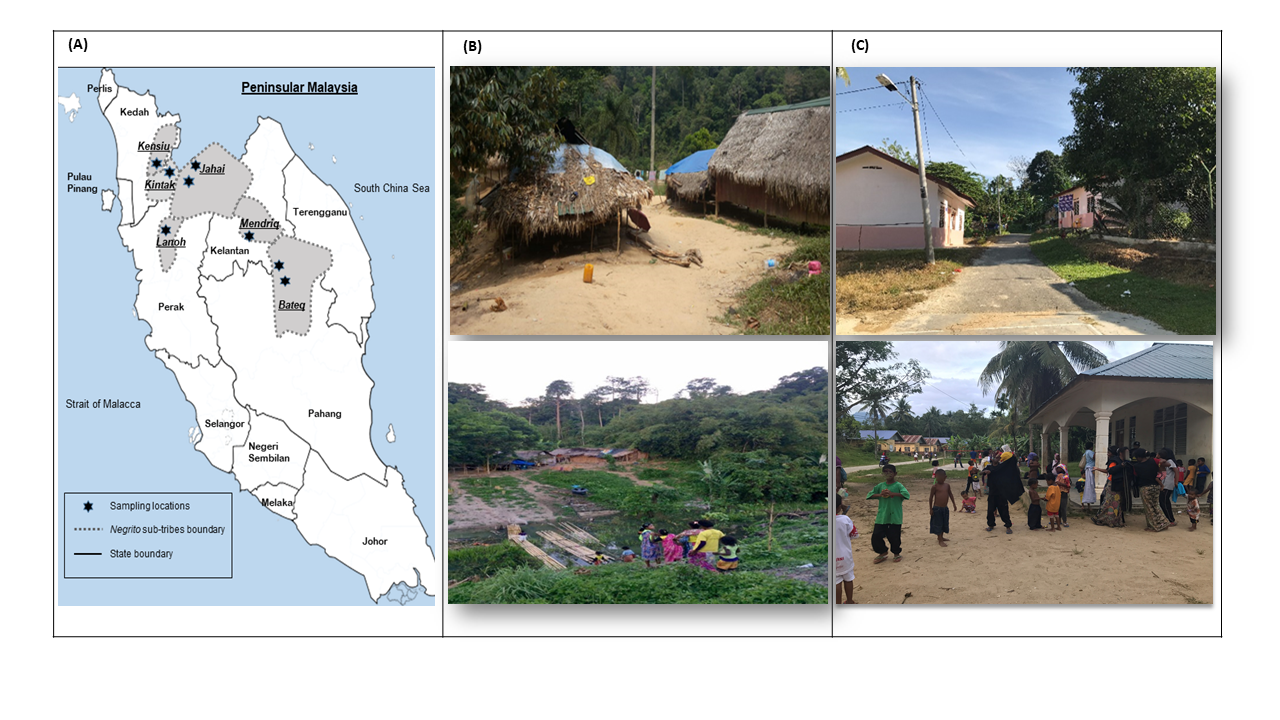

Supplement: S1 Fig — (A) A map showing the sampling locations which involved all sub-tribes of the Negrito. The Negritos are concentrated mainly in the northern states of Peninsular Malaysia. (B) The Inland Jungle Villages (IJV)—In situ improvement. (C) The Resettlement (RPS)—Ex situ development. Source: Map was recreated from a blank map of Peninsular Malaysia available at https://publicdomainvectors.org/en/free-clipart/Blank-map-of-peninsular-Malaysia/50795.html. (TIF) [file pntd.0007331.s002.tif]

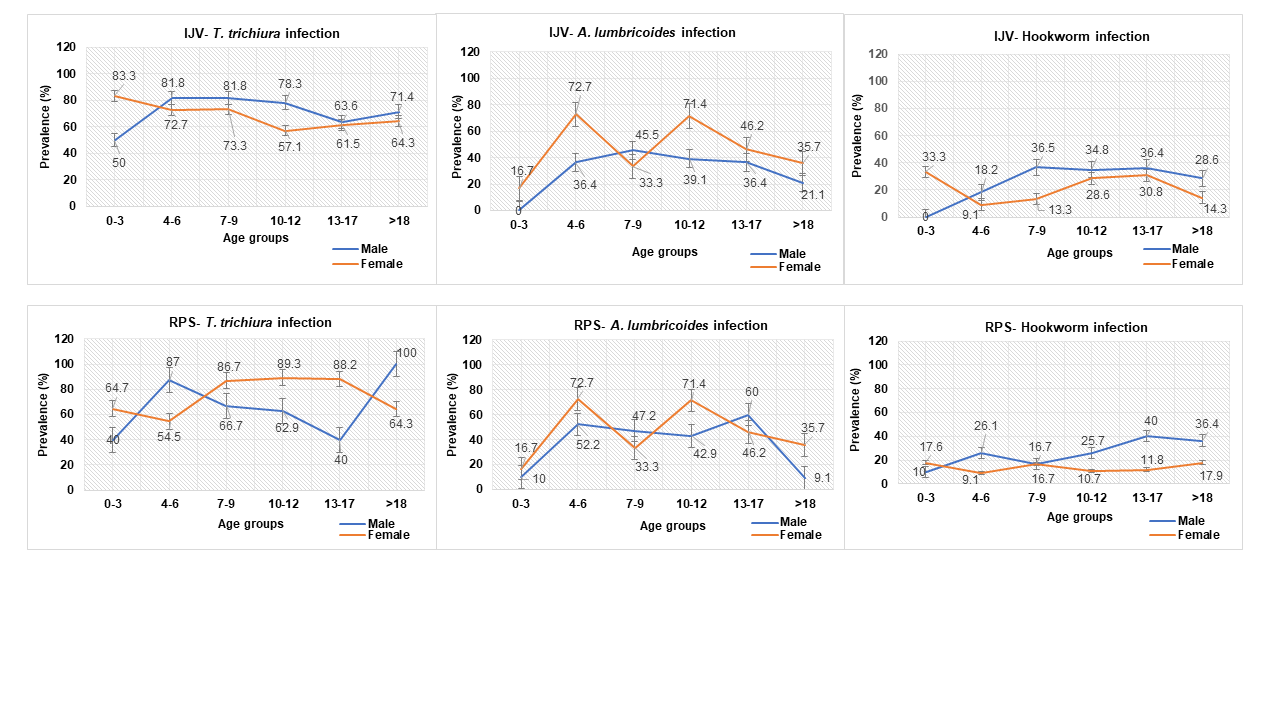

Supplement: S2 Fig — The females tend to be infected by STH at an early age of life compared to the male Negritos in both communities. In RPS, 100% positivity rate of T. trichiura infection was observed among the adult males, most likely because the targeted group for AHT treatment previously was pre- and school aged children. The rate of hookworm infection was higher in adult males and increased with age. (TIF) [file pntd.0007331.s003.tif]
